# Use of artificial intelligence in predicting in-hospital cardiac and respiratory arrest in an acute care environment—implications for clinical practice

**DOI:** 10.3389/fmedt.2025.1681059

**Published:** 2025-10-10

**Authors:** Geerthy Thambiraj, George Bazoukis, Amir Ghabousian, Jiandong Zhou, Sandeep Chandra Bollepalli, Eric M. Isselbacher, Vivian Donahue, Jagmeet P. Singh, Antonis A. Armoundas

**Affiliations:** ^1^ Cardiovascular Research Center, Massachusetts General Hospital, Boston, MA, United States; ^2^ Department of Cardiology, Larnaca General Hospital, Larnaca, Cyprus; ^3^ Medical School, European University Cyprus, Nicosia, Cyprus; ^4^ Department of Emergency Medicine, Massachusetts General Hospital, Boston, MA, United States; ^5^ Division of Health Science, Warwick Medical School, University of Warwick, Coventry, United Kingdom; ^6^ Healthcare Transformation Lab, Massachusetts General Hospital, Boston, MA, United States; ^7^ Cardiac Surgical Intensive Care Unit, Massachusetts General Hospital, Boston, MA, United States; ^8^ Cardiology Division, Cardiac Arrhythmia Service, Massachusetts General Hospital, Boston, MA, United States; ^9^ Broad Institute, Massachusetts Institute of Technology, Cambridge, MA, United States

**Keywords:** machine learning, artificial intelligence, cardiac arrest, respiratory arrest, intensive care unit

## Abstract

**Background:**

Artificial intelligence (AI)-based models can augment clinical decision-making, including prediction, diagnosis, and treatment, in all aspects of medicine.

**Research questions:**

The current systematic review aims to provide a summary of existing data about the role of machine learning (ML) techniques in predicting in-hospital cardiac arrest, life-threatening ventricular arrhythmias, and respiratory arrest.

**Methods:**

The study was conducted in compliance with the Preferred Reporting Items for Systematic Reviews and Meta-analyses (PRISMA) framework. PubMed, Embase, and Web of Science without any restriction were searched to extract relevant manuscripts until October 20, 2023. Additionally, the reference list of all potential studies was searched to identify further relevant articles. Original publications were regarded as eligible if they only recruited adult patients (≥18 years of age), employed AI/ML algorithms for predicting cardiac arrest, life-threatening ventricular arrhythmias, and respiratory arrest in the setting of critical care, used data gathered from wards with critically ill patients (ICUs, cardiac ICUs, and emergency departments), and were published in English. The following information was extracted: first author, journal, ward, sample size, performance and features of ML and conventional models, and outcomes.

**Results:**

ML algorithms have been used for cardiac arrest prediction using easily obtained variables as inputs. ML algorithms showed promising results (AUC 0.73–0.96) in predicting cardiac arrest in different settings, including critically ill ICU patients, patients in the emergency department and patients with sepsis, they demonstrated variable performance (AUC 0.54–0.94) in predicting respiratory arrest in COVID-19 patients, as well as other clinical settings.

**Conclusion:**

ML algorithms have shown promising results in predicting in-hospital cardiac and respiratory arrest using readily available clinical data. These algorithms may enhance early identification of high risk patients and support timely interventions, thereby reducing mortality and morbidity rates. However, the prospective validation of these algorithms and their integration into clinical workflows need further exploration.

## Introduction

Approximately 200,000 in-hospital cardiac (CA) and respiratory arrests (RA) occur annually in US hospitals (1, 2); survival is ∼25%, and has improved only moderately over recent decades (3–5). Identification of patients at risk for adverse events leading to CA has been key to improving outcomes. Despite numerous efforts, including early warning scores and rapid response protocols (6–11), recognizing high-risk patients remains a limiting step in providing pre-emptive care. Detection of patient deterioration typically occurs during clinical examination or vital sign measurements at varying intervals (12, 13), depending on hospital and intensive care unit (ICU) policy (14), which leaves significant potential for unnoticed patient deterioration (15, 16).

**Figure 1:**
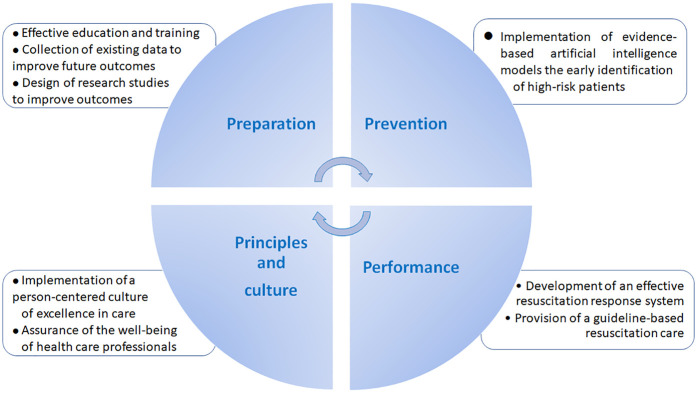
Practical measures to improve the outcomes of cardiac and respiratory arrest in clinical practice.

Given the potential culmination in mortality and serious neurological sequelae, timely detection of clinical deterioration is essential (17). While current risk-stratification tools, such as Early Warning Score (EWS) based methods, have aided in clinical decision-making, they are limited in accuracy, sensitivity, and user dependency (18). Accordingly, further improvements in the performance of predictive tools are warranted for better clinical judgment regarding in-hospital patient safety (19, 20) (Figure 1).

Artificial intelligence (AI)-based models can facilitate clinical decision-making (21–28) via handling of complex massive datasets (29–31). Considering the growing number of AI-based algorithms developed for predicting life-threatening events (32–34), the current systematic review aims to assess the role of machine learning (ML) algorithms in predicting cardiac arrest, life-threatening ventricular arrhythmias, and RA, in in-hospital, critically ill patients.

## Methods

The current systematic review study was conducted in compliance with the Preferred Reporting Items for Systematic Reviews and Meta-analyses (PRISMA) framework. This review was not registered and no protocol was prepared.

### Eligibility criteria

This review focuses on peer-reviewed articles that applied AI/ML methods to predict the occurrence of cardiac arrest, life-threatening ventricular arrhythmias (ventricular fibrillation, ventricular tachycardia, asystole, pulseless electrical activity), and RA in critical care settings. Original publications were regarded as eligible if they only recruited adult patients (≥18 years of age), employed AI/ML algorithms for predicting the above-mentioned adverse events, used data gathered from wards with critically ill patients (ICUs, cardiac ICUs, and emergency departments), and were published in English. Publications were excluded if they used data from general hospital wards. Apart from original articles, other journal manuscript types were excluded. Studies involving animals, *in vitro*, and *in vivo* research projects were also excluded. Out-of-hospital cardiac arrest patients were not included in this review.

### Search strategy

The research databases, including PubMed, Embase, and Web of Science, without any restriction, were used to extract relevant manuscripts until October 20, 2023. Moreover, the reference list of all potential studies was scrutinized and searched for additional articles. An advanced search strategy was conducted, structured around three groups of terms: critical care settings, artificial intelligence/machine learning, and cardiac or RA. Each group was searched using both exploded Emtree terms and keywords in titles, abstracts, and keyword fields. Terms within each group were combined using OR, and the three groups were combined using AND, ensuring retrieval of articles containing terms from all groups. Results were limited to publication types “Article”, “Article in Press”, and “Preprint”. A detailed search strategy is included in the Online Supplement.

### Data extraction

First, the identified citations from each database were uploaded into Endnote 20 and duplicates were eliminated. Two independent authors (AG, GB) screened the titles and abstracts of the remaining papers. Then, the selected full-text articles were reviewed according to the eligibility criteria in the same manner. Disagreements at any step were settled through discussion. The following information was extracted: first author name, journal, ward, sample size, performance and features of ML and conventional models, and outcomes.

A brief description of the reported AI/ML models in this manuscript is provided in the Online Supplement.

### Quality assessment

Risk of bias and quality assessment were performed using the QUADAS-2 tool. Two categories, risk of bias and concerns regarding applicability, were assessed in the three domains of patient selection, index test, and reference standard. With the former being assessed in the domain of flow and timing, as well. For assessing the risk of bias, the following criteria were applied for each of the four domains: (1) when the answer to all questions is “yes”, the overall bias risk of the domain is “low”; (2) when the answer to more than one question is “no”, bias risk was definitely identified, and the overall bias risk of the domain is “high”; (3) deemed “unclear” when the data reported is insufficient to make a judgment; (4) when any domain is high risk, the overall bias risk score is “high”; (5) only when the bias risk of one domain is unclear, the overall bias risk of the study is “unclear”.

The recommendation of the QUADAS-2 tool was followed, and the clinical applicability of each study was scored by evaluating whether it matched the concerns of our review, and rated as “low”, “high”, or “unclear”. An author (XL) independently performed the data extraction and quality assessment. Disagreements were resolved through discussion and independent assessment by another researcher to reach a consensus. The final study quality was classified as low risk of bias, high risk of bias, or unclear (Supplementary Table S1).

## Results

### Search results

**Figure 2:**
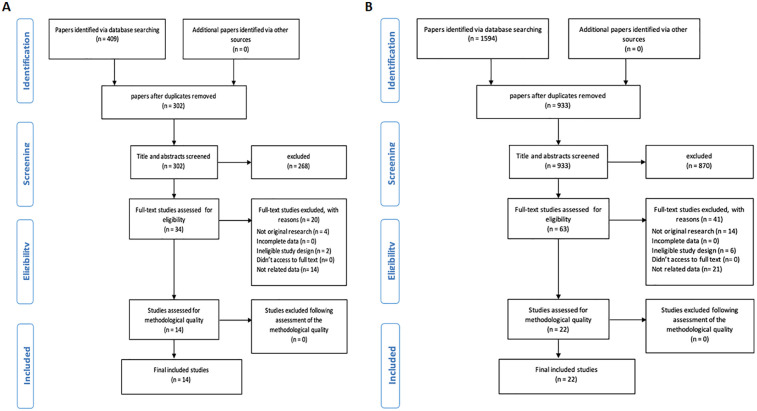
Flowchart of the search strategy. **(A)** Cardiac arrest, **(B)** Respiratory arrest.

Initially, we obtained 1,594 articles for RA and 409 for CA from three distinct databases, including PubMed, Embase, and Web of Science. Subsequently, we identified and removed duplicates (107 for CA, 661 for RA), leaving us with 302 CA articles and 933 RA articles. Finally, 14 CA studies and 22 RA studies met the inclusion and exclusion criteria and were included in the systematic review (Figures 2A,B, for CA and RA, respectively).

### Cardiac arrest

Prediction of cardiac arrest holds great importance in clinical practice in order to activate timely preventive measures. ML algorithms have been used to predict cardiac arrest using easily obtained variables as inputs (Table 1).

**Table 1:** Prediction of cardiac arrest.

Author	Journal	Data collection ward	Sample size	Outcome	Study results		Conclusion	Title
					AI/ML	Conventional methods		
Itai Bendavid	Scientific Reports	ICU	Step1 = 11,816 (MIMIC-III) Validation cohort = 1,061	Onset of invasive MV in hypoxemic patients with COVID-19	A two-step model 1: a XGBoost algorithm trained on non-COVID-19 critically ill patients, (AUC = 0.91, F1 = 0.19) 2: applying a transfer learning and adapting a model to a small group of COVID-19 patients, (AUC = 0.97, F1 = 0.09)		The model had good predictive ability. However, there was no comparison with previously existed models. Although the overall precision was low, the precision of prediction improved as prediction probability was higher.	A novel machine learning model to predict respiratory failure and invasive mechanical ventilation in critically ill patients suffering from COVID-19
Jeongmin Kim	Journal of Clinical Medicine	ICU	29,181	Prediction of respiratory failure	FAST-PACE AUC = 0.869 for respiratory failure	MEWS and NEWS scores	Artificial intelligence consisting of simple vital signs and a brief interview could predict acute respiratory failure 6 h earlier	Predicting Cardiac Arrest and Respiratory Failure Using Feasible Artificial Intelligence with Simple Trajectories of Patient Data
Huan Wang	BMC Pulmonary Medicine	ICU	929	Prediction of noninvasive ventilation failure	AUC CatBoost = 0.85 XGBoost = 0.81 GBDT = 0.81 RF = 0.80 COX = 0.76 LightGBM = 0.74 AdaBoost = 0.72 KNN = 0.70 LR = 0.68 NaiveBayes = 0.67 ML*P* = 0.66 SVM = 0.65		The CatBoost model can be used to identify patients who are at risk of noninvasive ventilation failure	Early prediction of noninvasive ventilation failure after extubation = development and validation of a machine-learning model
Jordan H. Chamberlin	Acad Radiol	ED	241	Prediction of intubation	Accuracy of artificial intelligence airspace opacity ≥12 for intubation = 0.839		The tested deep convolutional neural network can help clinicians to predict outcomes in COVID-19 patients.	An Interpretable Chest CT Deep Learning Algorithm for Quantification of COVID-19 Lung Disease and Prediction of Inpatient Morbidity and Mortality
Limin Yu	PlonOne	ED	1,036	Prediction of mechanical ventilation	ACC = 86.2 NPV = 87.8%		XGBoost algorithm can predict the need for mechanical ventilation in COVID-19 patients.	Machine learning methods to predict mechanical ventilation and mortality in patients with COVID-19
Xue Feng	Computer Methods and Programs in Biomedicine	ICU	5,653	Prediction of noninvasive ventilation failure	AUC Time Updated Light Gradient Boosting Machine (TULightGBM) = 0.8323	AUC LR = 0.6960 RF = 0.8039 XGBoost = 0.8301 ANN = 0.7533 LSTM = 0.8158	The TULightGBM model can be used to predict the late noninvasive ventilation failure with high accuracy	Dynamic prediction of late noninvasive ventilation failure in intensive care unit using a time adaptive machine model
Alberto Di Napoli	Journal of Digital Imaging	ED	1,051	Prediction of intubation	Neural Network ACC = 70,7% SEN = 72,3% SPE = 50%		3D CT-based deep learning model showed a good performance in predicting intubation in COVID-19 patients	3D CT-Inclusive Deep-Learning Model to Predict Mortality, ICU Admittance, and Intubation in COVID-19 Patients
Patrick Essay	Respiratory Care	ICU	22,075	Prediction of noninvasive respiratory support failure	AUC LSTM = 0.963 GRU = 0.953 GRU-D = 0.79 RF = 0.796	AUC LR = 0.795	Recurrent neural network models can predict noninvasive respiratory support failure using routinely collected time series data	Predicting failure of noninvasive respiratory support using deep recurrent learning
Ming Xia	Ann Transl Med	ICU	14,777	Prediction of hypoxemia after extubation	AUC RF = 0.792 (95% CI, 0.771–0.814) LightGBM = 0.792 (95% CI, 0.770–0.815) KNN = 0.763 (0.739–0.786) Logistics Regressio*n* = 0.775 (0.751–0.799) SVM = 0.737 (0.713–0.761) Extreme Gradient Boosting = 0.717 (0.693–0.742)		ML models have considerable potential for predicting hypoxemia after extubation	Development and validation of a machine-learning model for prediction of hypoxemia after extubation in intensive care units
Benjamin Ming Kit Siu	Scientific Reports	ICU	17,616 (eICU-CRD and MIMIC-III)	Need for intubation within the first 24 h of ICU admission	RF: AUC = 0.86	LR: AUC = 0.77	RF models outperformed LR models in predicting need for intubation in critically ill patients based on bedside parameters.	Predicting the need for intubation in the first 24 h after critical care admission using machine learning approaches
Siavash Bolourani	Journal of Medical Internet Research	ED	11,525	Respiratory failure within 48 h of admission at ED	XGBoost: AUC = 0.77, ACC = 0.919 XGBoost + SMOTEENN = AUC = 0.76, ACC = 0.893	LR: AUC = 0.70, ACC = 0.915	ML models were superior to LR models in terms of precision and ACC.	A Machine Learning Prediction Model of Respiratory Failure Within 48 H of Patient Admission for COVID-19= Model Development and Validation
Frank S. Heldt	Scientific Reports/2021	ED	Invasive ventilation cohort = 878	Need for MV in COVID-19 patients using ED data	XGBoost: AUC = 0.87, F1 = 0.42 RF: AUC = 0.87, F1 = 0.31	LR: AUC­ = 0.74, F1 = 0.23	ML models were superior to conventional models	Early risk assessment for COVID-19 patients from emergency department data using machine learning
Adrian D. Haimovich	Annals of Emergency Medicine	ED	1,172	Respiratory deterioration within the first 24 h of hospitalization (requiring oxygen ≥ 10 L/min, noninvasive ventilation, or intubation.)	ML with gradient boosting: AUC = 0.76, ACC = 0.79, Brier score = 0.25, F1 = 0.47, Precision = 0.4	Bootstrapped LR models: qSOFA = AUC = 0.59, ACC = 0.83, Brier = 0.12, F1 = 0.08, Precision = 0.20 CURB-65: AUC = 0.50, ACC = 0.64, Brier = 0.12, F1 = 0.13, Precision = 0.16 Elixhauser: AUC = 0.61, ACC = 0.49, Brier = 0.12, F1 = 0.28, Precision = 0.20 qCSI: AUC = 0.81, ACC = 0.82, Brier = 0.10, F1 = 0.49, Precision = 0.44	The qCSI and a machine-learning model (CSI) outperformed the Elixhauser mortality index, CURB-65, and qSOFA. CSI performance on the validation cohort was not superior to that of the qCSI.	Development and Validation of the Quick COVID-19 Severity Index = A Prognostic Tool for Early Clinical Decompensation
Finneas J.R. Catling	Journal of the American Medical Informatics Association	ICU	4,713	Prediction of intubation	AUC Temporal Convolutional Networks FFNN = 0.903 LSTM-FFNN = 0.882 FFNN = 0.858	AUC LR = 0.847	Temporal convolutional networks improve prediction of clinical events	Temporal convolutional networks allow early prediction of events in critical care
Michaela Venturinin	AIME	ICU	4,130	Prediction of intubation	AUC Cure-ML = 0.80	AUC Standard survival model (RSF) = 0.76 Random forest = 0.77	The proposed model can improve the prediction of the need for intubation in critically ill patients by using routinely collected data	A Novel Survival Analysis Approach to Predict the Need for Intubation in Intensive Care Units
Renata G Mendes	Arch Med Sci	ED	1,315	Prediction of reintubation and prolonged mechanical ventilation	ANN Reintubation ACC = 0.63 SEN = 0.64 SPE = 0.63 Prolonged mechanical ventilation ACC = 0.63 SEN = 0.76 SPE = 0.62	LR Reintubation ACC = 0.60 SEN = 0.64 SPE = 0.60 Prolonged mechanical ventilation ACC = 0.63 SEN = 0.64 SPE = 0.63	The ANN has similar discriminating power in predicting reintubation and prolonged mechanical ventilation	Predicting reintubation, prolonged mechanical ventilation and death in post-coronary artery bypass graft surgery = a comparison between artificial neural networks and logistic regression models
Salah Boussen	Computers in Biology and Medicine	ICU	279	Need for intubation in COVID-19 ICU patients based on SpO_2_ and breathing frequency		Unsupervised clustering: AUC = 0.94, ACC = 0.878 TPR = 86.5%, TNR = 90.9%	The algorithm was able to successfully categorize patients according to their risk of intubation.	Triage and monitoring of COVID-19 patients in intensive care using unsupervised machine learning
Roshan Karri	PLOS ONE	ICU	300	Need for MV within the first three days of admission in ICU patients with COVID-19	KNN: AUC = 0.59, SEN = 0.78, SPE = 0.49 DT: AUC = 0.54, SEN = 0.31, SPE = 0.78 SVM: AUC = 0.65, SEN = 0.78, SPE =0.59 GBM: AUC = 0.68, SEN = 0.81, SPE = 0.58 RF: AUC = 0.69, SEN = 0.77, SPE = 0.62	LR: AUC = 0.64, SEN = 0.75, SPE = 0.59	RF and GBM were the best performing algorithms for predicting the short-term requirement for invasive ventilation.	Machine learning can be used to predict the short-term requirement for invasive ventilation among Australian critically ill COVID-19 patients
Dan Assaf	Internal and Emergency Medicine	ED	162	Progression of non-critical COVID-19 patients to respiratory failure , hospitalization in ICU multi-organ failure and/or death	ANN: AUC = 0.92, SEN = 0.59, SPE = 0.96, ACC = 0.91 RF: AUC = 0.93, SEN = 0.75, SPE = 0.96, ACC = 0.93 CRT: AUC = 0.90, SEN = 0.88, SPE = 0.93, ACC = 0.92	APACHE II: AUC = 0.79, SEN = 0.68, SPE = 0.81, ACC = 0.79	ML algorithms amplify the diagnostic accuracy and the discriminative efficacy of routinely used markers and scoring systems.	Utilization of machine-learning models to accurately predict the risk for critical COVID-19
Supreeth P. Shashikumar	Chest	ICU	22,416	Onset of mechanical ventilation of ICU patients 24 h in advance	VentNet (a two-layer feedforward neural network of size 40 and 25): AUC = 0.895 (general ICU patients), AUC = 0.944 (COVID-19 patients)	LR: AUC = 0.769 (general ICU patients), AUC = 0.786 (COVID-19 patients) ROX score: AUC = 0.738 (general ICU patients), AUC = 0.849 (COVID-19 patients)	A high-performing deep learning model (AUC > 0.88) can predict future need for MV 24 h in advance using commonly accessible EHR data	Development and Prospective Validation of a Deep Learning Algorithm for Predicting Need for Mechanical Ventilation
Mariana Frizzo de Godoy	Radiol Bras	ICU	937	Prediction of mechanical ventilation	SEN = 0.417 SPE = 0.860 AUC = 0.68		The deep learning model can reliably predict which patients will require invasive ventilation	Artificial intelligence to predict the need for mechanical ventilation in cases of severe COVID-19
Huiquan Wang	Biomedical Signal Processing and Control	ICU	1,613	Prediction of mechanical ventilation	AUC Lightgbm = 0.917	AUC RF = 0.907 LR = 0.888 Naïve Bayes = 0.887 MLP = 0.883 SVM = 0.843 KNN = 0.774	The proposed real-time warning model can be used to predict the need for mechanical ventilation	Invasive mechanical ventilation probability estimation using machine learning methods based on non-invasive parameters
Nan Liu	BMC Medical Informatics and Decision Making	ED	702	Predicting major adverse cardiac events (death, cardiac arrest, sustained Ventricular tachycardia, and hypotension) using clinical signs and heart rate variability in chest pain patients within 72 h of arrival	ML based on 3 most relevant variables= AUC = 0.81, SEN = 0.82, SPE = 0.63 ML based on 23 (all) variables= AUC = 0.73, SEN = 0.72, SPE = 0.63	TIMI: AUC = 0.63 MEWS = AUC = 0.62	The proposed ML scoring system outperformed traditionalrisk stratification systems such as TIMI and MEWS.	RF-based method was developed to select the most relevant variables. A geometric distance-based ML scoring system was then implemented to derive a risk score.

ACC, accuracy; ANN, artificial neural network; AUC, area under the curve; AUPRC, area under precision recall; CA, cardiac arrest; CRT, classification and regression decision tree; DT, decision tree; ED, emergency department; EHR, electronic health record; FPR, false positive rate; GBM, gradient boosting machine; ICU, intensive care unit; KNN k-nearest neighbor; LR, logistic regression; MEWS, the modified early warning score; MLP, multilayer perceptron; ML, machine learning; NEWS, national early warning score; NPV, negative predictive value; PPV, positive predictive value; RF, random forest; SEN, sensitivity; SPE, specificity; SVM, support vector machine; TCM, temporal convolutional network; LSTM, long short-term memory.

Intensive Care Unit Yijing L et al., studied a cardiac arrest prediction index in critically ill ICU patients (35). In this study, bedside vital signs monitoring was used as inputs (heart rate, systolic blood pressure, diastolic blood pressure, mean blood pressure, SpO_2,_ and respiratory rate) (35). The cardiac arrest prediction index predicted 95% of cardiac arrest events. Interestingly, 80% of the cardiac arrest events were identified more than 25 min in advance (35). In a study by Kim J et al., the authors used bedside vital signs, underlying disease, laboratory data, medication, and organ failure to predict cardiac arrest in critically ill patients using ML models (36). The proposed model showed a sensitivity between 0.846 and 0.909, and a specificity between 0.923 and 0.946 (36).

Another deep learning model has been proposed for cardiac arrest prediction in ICU patients using physiological and demographic features. The proposed model outperformed the Modified Early Warning Score (MEWS) and National Early Warning Score (NEWS) scores in cardiac arrest prediction at the tested time intervals (17). Tang Q et al., proposed another deep learning model based on time series of vital signs from electronic health records. In this model, features were captured by an efficient temporal convolutional network and explained using the deep Taylor decomposition theoretical framework. The results showed that the model demonstrated superior CA prediction accuracy compared to the standard NEWS score (37). An artificial neural network (ANN) has been developed to predict ventricular tachycardia 1 h before its onset, using parameters obtained from heart rate variability and respiratory rate variability analysis (38). The ventricular tachycardia prediction model achieved a sensitivity of 88%, specificity of 82%, and an AUC of 0.93 (38).

#### Emergency department

Another topic of interest is the prediction of in-hospital CA in patients who presented to the emergency department. In this setting, a ML model has been implemented using triage data. The authors showed that Random Forest outperformed other ML models (Gradient Boosting and Extra Trees classifier), achieving an AUC of 0.931 (39). Interestingly, although the difference in AUC between each ML model and logistic regression was not significant, ML models performed significantly better than the NEWS scoring system (39). An ML algorithm has also been proposed to predict critical care outcomes, including CA, in patients with chest pain presenting to the emergency department (40). Specifically, a LASSO regression model was developed using easily obtained features. The proposed model significantly outperformed the HEART, GRACE, and TIMI scores achieving an AUC of 0.953 (95% CI: 0.922–0.984) (40). Liu N et al., aimed to identify the most relevant variables for predicting major adverse cardiac events including CA, in patients presented to the emergency department (41). The authors used a novel random forest-based method to select the most relevant variables while a geometric distance-based ML scoring system was implemented to derive the risk score. The use of three variables (systolic blood pressure, the mean electrocardiographic RR interval and the mean instantaneous heart rate) demonstrated good performance in predicting adverse events (AUC: 0.812), outperforming the model using 23 variables (AUC: 0.736), and the conventional TIMI (AUC: 0.637) and MEWS (AUC: 0.622) scores (41).

An ML model incorporating heart rate variability was proposed to predict CA in critically ill patients presenting to the emergency department (42). The results showed that the ML model outperformed the conventional methods in predicting CA within 72 h, with an AUC of 0.781 compared to 0.680 for MEWS (42). ML models developed on triage data have also been proposed to predict in-hospital CA or ICU admissions in patients visiting the emergency department (43). The proposed model demonstrated better sensitivity and accuracy in predicting critical outcomes compared to the assessments made by emergency physicians (43).

#### Sepsis

ML models have been implemented for the prediction of CA in patients with sepsis. In this setting, the best results were obtained using a stacking algorithm and multivariate dataset (44). The proposed model predicted the arrest incidence with an accuracy and sensitivity of over 70%, up to 6 h earlier. Although ML algorithms outperformed the conventional methods (APACHE II and MEWS scoring variables) for determining the patients' health status, higher sensitivity and specificity are needed for implementation in clinical practice (44). Baral S et al., proposed a deep learning algorithm to reduce the false alarm rates and increase the sensitivity of the previous models for CA prediction in patients with sepsis (45). Specifically, a hybrid model using a multilayer perceptron and enhanced bidirectional Long Short-Term Memory (LSTM) was proposed to handle baseline features and time-series vital signs (45). Compared to the state-of-the-art algorithms, the proposed model improved accuracy, sensitivity, specificity, and AUC, while reducing the false alarm rates.

### Respiratory arrest

Prediction of RA and the need for mechanical ventilation can help clinicians identify high-risk patients and implement timely preventive measures (Table 2).

**Table 2:** Prediction of respiratory arrest.

Author	Journal	Data collection Ward	Sample size	Outcome	Study results		Conclusion	AI methods
					AI/ML	Conventional methods		
Li Yijing	Computer Methods and Programs in Biomedicine	ICU (MIMIC-III database)	1,860 patients (169 CA patients and 1,691 non-CA patients)	The risk of developing CA within the next 1 h, every 5 min, based on the features generated from previous 2 h data.	Test set: AUC = 0.94 SEN = 0.86 SPE = 0.85 ACC = 0.96 Identifying CA patients (SEN = 95%) F1 = 0.05 non-CA patients: Error rate = 37%, SPE = 0.63		The model can aid in predicting CA via vital signs monitoring. False positive prediction was relatively high (37%), which mandates employing a false alarm reduction strategy.	Extreme gradient boosting (XG- Boost) three-fold cross validation
Junetae Kim	JMIR Medical Informatics	ICU	759 patients (37 CA patients and 722 non-CA patients) 80% model development 20% testing	The risk of CA in critically ill patients	Median value of the performance of the 5-cross validation set: 1 h before CA: AUC = 0.96, ACC = 0.97, SEN = 0.88, SPE = 0.97 4 h before CA: AUC = 0.94, ACC = 0.98, SEN = 0.85, SPE = 0.98 48 h before CA = AUC = 0.74, ACC = 0.57, SEN = 0.92, SPE = 0.55		The model performance decreased in accordance with increasing time to event.	Deep learning (Character-level gated recurrent unit with a Weibull distribution algorithm) Fivefold cross-validation
Tsung-Chien Lu	Internal and Emergency Medicine	ED	316,465 patients	Predicting loss of a palpable pulse with attempted resuscitation in the ED	RF: AUC = 0.93 (95% CI 0.91–0.95), ACC = 0.92, SEN = 0.75, SPE = 0.92, precision = 0.019, Brier = 0.066, AUPRC = 0.116 GB: AUC = 0.93 (95% CI 0.91–0.95), ACC = 0.93, SEN = 0.74, SPE = 0.93, precision = 0.02, Brier = 0.07, AUPRC = 0.057 ET: AUC = 0.92 (95% CI 0.89–0.94), ACC = 0.91, SEN = 0.76, SPE = 0.91, precision = 0.017, Brier = 0.075, AUPRC = 0.045	LR: AUC = 0.91 (95% CI = 0.88–0.93), ACC = 0.998, SEN = 0.00, SPE = 1.00, precision = 0.00, Brier = 0.002, AUPRC = 0.026 NEWS2 scoring system (compared to ML/LR = *P* < 0.05): AUC = 0.678 (95% CI 0.675–0.681), ACC = 0.91, SEN = 0.26, SPE = 0.91, precision = 0.006, AUPRC = 0.004	ML models statistically significantly outperformed NEWS2 scoring system. However, the differences between each of ML models and LR were not significant.	Supervised ML algorithms using RF, Gradient Boosting (GB), and Extra Trees (ET) classifiers nine-fold cross validation
Jeongmin Kim	Journal of Clinical Medicine	ICU	27,708 patients	Predicting CA and respiratory failure in ICU patients in a real-world setting	DL: 1 h before CA: AUC = 0.896, SEN = 0.84, SPE = 0.78, PPV = 0.10, NPV = 0.99 ACC = 0.78 F2-score = 0.178 2 h before CA: AUC = 0.89, SEN = 0.87, SPE = 0.74, PPV = 0.09, NPV = 0.995 ACC = 0.75, F2-score = 0.16 6 h before CA: AUC = 0.89, SEN = 0.86, SPE = 0.75 PPV = 0.08, NPV = 0.995, ACC = 0.75 F2-score = 0.15	MEWS: 1 h before CA: AUC = 0.75, SEN = 0.41, SPE = 0.88, PPV = 0.09, NPV = 0.98, ACC = 0.86, F2-score = 0.15 2 h before CA: AUC = 0.75, SEN = 0.41 SPE = 0.88 PPV = 0.09, NPV = 0.99, ACC = 0.86 F2-score = 0.14 6 h before CA: AUC = 0.74, SEN = 0.39, SPE = 0.88, PPV = 0.08, NPV = 0.98, ACC = 0.86, F2-score = 0.13 NEWS: 1 h before CA: AUC = 0.76, SEN = 0.70, SPE = 0.71, PPV = 0.07, NPV = 0.99, ACC = 0.71, F2-score = 0.12 2 h before CA: AUC = 0.76, SEN = 0.70, SPE = 0.71, PPV = 0.06, NPV = 0.99, ACC = 0.71, F2-score = 0.12 6 h before CA: AUC = 0.75, SEN = 0.69, SPE = 0.71, PPV = 0.06, NPV = 0.99, ACC = 0.71, F2-score = 0.10	DL model achieves statistically significant higher performance in terms of AUC than MEWS or NEWS for CA prediction 1–6 h before the incident.	Deep learning
Qinhua Tang	Mathematical biosciences and engineering	ICU (MIMIC-III database)	486 patients 107 CA positive	Predicting CA in ICU patients	RF: 1 h before CA: AUC = 0.75, SEN = 0.67 SPE = 0.87, PPV = 0.57, NPV = 0.91, F1-score = 0.62. 2 h before CA: AUC = 0.75, SEN = 0.73, SPE = 0.86, PPV = 0.52, NPV = 0.94, F1-score = 0.61. 5 h before CA: AUC = 0.75, SEN = 0.61 SPE = 0.86, PPV = 0.52, NPV = 0.89, F1-score = 0.56 GRU: 1 h before CA: AUC = 0.76, SEN = 0.53, SPE = 0.90, PPV = 0.78, NPV = 0.74, F1-score = 0.63. 2 h before CA: AUC = 0.75, SEN = 0.53, SPE = 0.87, PPV = 0.71, NPV = 0.76, F1-score = 0.61. 5 h before CA: AUC = 0.69, SEN = 0.44, SPE = 0.87, PPV = 0.75, NPV = 0.63, F1-score = 0.55 LSTM: 1 h before CA: AUC = 0.83, SEN = 0.55 SPE = 0.98, PPV = 0.96, NPV = 0.71, F1-score = 0.70. 2 h before CA: AUC = 0.77, SEN = 0.53, SPE = 0.93, PPV = 0.88, NPV = 0.69, F1-score = 0.66. 5 h before CA: AUC = 0.76, SEN = 0.50 SPE = 0.89, PPV = 0.80, NPV = 0.67, F1-score = 0.62 TCN model: 1 h before CA:AUC = 0.85, SEN = 0.75 SPE = 0.90, PPV = 0.75, NPV = 0.90, F1-score = 0.75. 2 h before CA: AUC = 0.83, SEN = 0.71, SPE = 0.87, PPV = 0.68, NPV = 0.88, F1-score = 0.69. 5 h before CA: AUC = 0.80, SEN = 0.68 SPE = 0.87, PPV = 0.68, NPV = 0.87, F1-score = 0.68	NEWS: 1 h before CA: AUC = 0.62, SEN = 0.62 SPE = 0.68, PPV = 0.38, NPV = 0.85, F1-score = 0.47 2 h before CA:AUC = 0.57, SEN = 0.55 SPE = 0.65, PPV = 0.33, NPV = 0.82, F1-score = 0.41 5 h before CA: AUC = 0.58, SEN = 0.53 SPE = 0.67, PPV = 0.34, NPV = 0.82, F1-score = 0.41	The TCM model achieved superior CA prediction ACC compared with NEWS, in terms of overall AUC and F1-Score. The model had an overall better performance compared to traditional DL models.	TCM (explained by Deep Taylor decomposition)
Samaneh Layeghian Javan	Computer Methods and Programs in Biomedicine	ICU (MIMIC-III database)	4,611 patients 79 CA cases 4,532	Predicting CA for adult patients with sepsis up to 6 h earlier	Best model using multivariate dataset: Stacking model (balanced by K-medoid) 1 h before CA: AUC = 0.82, SEN = 0.77, SPE = 0.76, ACC = 0.76, precision = 0.19, F1 score = 0.31, FPR = 0.24 Best model using time series: Kernel SVM (balanced by SMOTE) 1 h before CA: AUC = 0.81, SEN = 0.70, SPE = 0.76, ACC = 0.76, precision = 0.17, F1 score = 0.27, FPR = 0.24 Best model using combined dataset LR (balanced by weighting) 1 h before CA: AUC = 0.78, SEN = 0.70, SPE = 0.78, ACC = 0.77, precision = 0.18, F1 score = 0.28, FPR = 0.22	APACHE II 1 h before CA: AUC = 0.71, SEN = 0.67, SPE = 0.75, ACC = 0.74, precision = 0.18, F1 score = 0.28, FPR = 0.25 MEWS: 1 h before CA: AUC = 0.70, SEN = 0.62, SPE = 0.78, ACC = 0.77, precision = 0.20, F1 score = 0.30, FPR = 0.22	The best results were obtained using a stacking algorithm. The model produced a significant improvement in the SEN and AUC values compared to APACHE II and MEWS.	classical methods (SVM, DT, LR, KNN, Gaussian NB), and ensemble methods (gradient boosting, XGBoost, RF, balanced bagging classifier and stacking). Three datasets (multivariate, time series and combined) were created and compared in 6 different class-hour groups.
Samit Baral	Multimedia Tools and Applications	ICU	7,611 patients MIMIC III database	Prediction of cardiac arrest in patients with sepsis	Proposed solution ACC = 0.926, SENS = 0.943, SPE = 0.936 and AUC = 0.94	State of Art solution ACC = 0.857, SENS = 0.877, SPE = 0.849, and AUC = 0.86	The proposed system is reducing the false alarm rate and increasing accuracy, sensitivity, specificity, and the area under curve for the prediction of cardiac arrest using enhanced Bidirectional LSTM model	MLP Enhanced Bidirectional LSTM
Ting Ting Wu	BMC Emergency Medicine	ED	483 patients (71 CA patients, 138 ICU admission, 10 mortality)	predicting critical care outcomes (CA, ICU admission, death) in ED patients with chest pain	LASSO:ACC = 0.89, SEN = 0.86, SPE = 0.91, PPV = 0.89, NPV = 0.89, F1 = 0.88, AUC = 0.95	GRACE: ACC = 0.72, SEN = 0.61, SPE = 0.81, PPV = 0.73, NPV = 0.71, F1 = 0.66, AUC = 0.75 HEART: ACC = 0.71, SEN = 0.77, SPE = 0.66, PPV = 0.65, NPV = 0.78, F1 = 0.71, AUC = 0.75 TIMI: ACC = 0.68, SEN = 0.50, SPE = 0.84, PPV = 0.72, NPV = 0.67, F1 = 0.59, AUC = 0.74	The model significantly outperformed the HEART, GRACE, TIMI score	LASSO regression model
Marcus Eng Hock Ong	Critical Care	ED	925 patients 43 CA patients	Predicting CA in critically ill patients within 72 h of presentation to the ED	ML:AUC = 0.78, SEN = 0.81, SPE = 0.72, PPV = 0.13, NPV = 0.99	MEWS:AUC = 0.6, SEN = 0.74, SPE = 0.54, PPV = 0.0, NPV = 0.98	The ML model outperformed the MEWS	Multivariate, nonparametric blackbox approach
Dong-Hyun Jang	American Journal of Emergency Medicine	ED	374,605	Predicting the development of cardiac arrest within 24 h of ED presentation	*ΑΝΝ*-MLP: AUC = 0.929 (0.926–0.932) *ΑΝΝ*-LSTM:AUC = 0.933 (0.930–0.936) *ΑΝΝ*-Hybrid: AUC = 0.936 (0.933–0.939)	MEWS: AUC = 0.886 (0.882–0.891) LR: AUC = 0.914 (0.910–0.918) RF: AUC = 0.923 (0.919–0.926)	Although all models achieved high performance in terms of AUC, the ANN models statistically significantly outperformed non-ANN models (*P* < 0.001). The hybrid ANN model utilizing both baseline and sequence information achieved the best performance.	Three ANN models = MLP model, LSTM model, the hybrid model (baseline variables are processed via MLP and sequence data are processed via LSTM) compared to non-ANN models (RF, LR) and MEWS
Ji Hoon Kim	Scientific reports	ED	1,350,693 CA incidence = 0.4%	the occurrence of CA in the ED of patients arriving via EMS	LR:AUC = 0.91 XGB: AUC = 0.92 MLP:AUC = 0.91		The machine-learning predictive model using the integrated information acquired in the prehospital stage effectively predicted in-hospital cardiac arrest in the ED.	LR, extreme gradient boosting (XGB, XGBoost), and MLP
Min-Chen Chen	Journal of Biomedical Informatics	ED	171,275	Predicting critical outcomes (in-hospital cardiac arrest and ICU admission) based on the history and vital signs routinely collected at triage.	Proposed DL model: AUC = 0.87, SEN = 0.50, SPE = 0.93, PPV = 0.16, NPV = 0.99 BiLSTM + TR: AUC = 0.84, SEN = 0.45, SPE = 0.94, PPV = 0.16, NPV = 0.99 RF: AUC = 0.79, SEN = 0.12, SPE = 0.99, PPV = 0.22, NPV = 0.98	For 30 random visits: doctor's prediction: SEN = 0.41, SPE = 0.78, PPV = 0.47, NPV = 0.74, ACC = 0.67 Proposed deep learning model: SEN = 0.95, SPE = 0.77, PPV = 0.9, NPV = 0.87, ACC = 0.90	The model showed better sensitivity and accuracy in predicting critical outcomes than the emergency physicians and available AI methods.	Clinical narrative-aware deep learning approach
Hyojeong Lee	Scientific reports	ICU		Predicting VT one hour before its onset using parameters obtained from heart rate variability and respiratory rate variability analysis	ANN: SEN = 0.88, SPE = 0.82, ACC = 0.85, PPV = 0.83, NPV = 0.87, AUC = 0.93		employing both ECG and respiratory signals can increase the performance of detecting VT one hour before its occurrence.	ANN
Nan Liu	BMC Medical Informatics and Decision Making	ED	72	Predicting major adverse cardiac events (death, cardiac arrest, sustained Ventricular tachycardia, and hypotension) using clinical signs and heart rate variability in chest pain patients within 72 h of arrival	ML based on 3 most relevant variables: AUC = 0.81, SEN = 0.82, SPE = 0.63 ML based on 23 (all) variables: AUC = 0.73, SEN = 0.72, SPE = 0.63	TIMI: AUC = 0.63 MEWS: AUC = 0.62	The proposed ML scoring system outperformed traditional risk stratification systems such as TIMI and MEWS.	RF-based method was developed to select the most relevant variables. A geometric distance-based ML scoring system was then implemented to derive a risk score.

ACC, accuracy; ANN, artificial neural network; AUC, area under the curve; CRT, classification and regression decision tree; EHR, electronic health record; GBM, gradient boosting machine; ICU, intensive care unit; KNN k-nearest neighbor; LR, logistic regression; LSTM, long short-term memory; MEWS, the modified early warning score; MLP, multilayer perceptron; NEWS, national early warning score; NPV, negative predictive value; PPV, positive predictive value; RF, random forest; SEN, sensitivity; SPE, specificity; SVM, support vector machine.

#### COVID-19

The random forest classifier, decision tree classifier, logistic regression, K-nearest neighbors classifier, support vector machine, and gradient boosted machine have been used for the prediction of invasive ventilation in COVID-19 patients admitted to the ICU (46). The random forest and Gradient boosted machine showed the best performance, achieving mean AUCs of 0.69 and 0.68, respectively (46). In the same setting, commonly used clinical variables (heart rate, oxygen saturation, respiratory rate, FIO_2_, and pH) were used as inputs in a deep learning model for the prediction of mechanical ventilation in hospitalized patients and in those with COVID-19 (47). The proposed model showed good performance (AUC > 0.88) in predicting those needing mechanical ventilation 24 h in advance (47). In addition, a two-step model has been used for the prediction of respiratory failure and invasive mechanical ventilation in critically ill patients suffering from COVID-19 (48). An Extreme Gradient Boosting (XGBoost) algorithm was trained on data from the MIMIC-III database to predict if a patient would require invasive mechanical ventilation within the next 6, 12, 18 or 24 h. The proposed two-step model showed good performance in both the general ICU population and COVID-19 patients (48).

A 3D CT-based deep learning model has also been proposed for the prediction of COVID-19 outcomes, including the need for intubation (49). The prediction results improved when laboratory data were included, while the model accuracy decreased when CT images were excluded (49). A deep convolutional neural network (dCNN) was evaluated to predict inpatient outcomes, including intubation associated with COVID-19 pneumonia (50). Airspace opacity scoring systems, defined by the extent of airspace opacity in each lobe on chest CT scans, were estimated using the deep learning algorithm and used to predict clinical outcomes. Τhe tested algorithm was found to be highly predictive of inpatient outcomes, including intubation (50). De Godoy MF et al., studied the role of CT imaging, assessed by dCNN, in predicting the need for mechanical ventilation in the setting of COVID-19 (51). The high specificity exhibited by the model enabled it to predict which patients may need mechanical ventilation due to COVID-19 infection (51). Bussen S et al., used an unsupervised ML algorithm (the Gaussian mixture model) to predict intubation in COVID-19 patients (52). The algorithm achieved an accuracy of 87.8% for intubation recognition using simple parameters (breathing frequency and SpO_2_) (52). In addition, XGBoost and Categorical Boosting (CatBoost) algorithms demonstrated high accuracy in predicting the need for mechanical ventilation in COVID-19 patients, using vital signs and demographics for initial triage, in the emergency department (53). In another study, XGBoost and Random Forest outperformed Logistic regression in predicting mechanical ventilation in COVID-19 patients using electronic health records data, in the emergency department (54). Similarly, another study showed that the XGBoost model had the highest mean accuracy for predicting respiratory failure within 48 h of a patient's admission for COVID-19 (55). XGBoost outperformed SMOTEENN XGBoost, Logistic regression, and the Modified Early Warning Score (55). Easily obtained variables were used as inputs including the type of oxygen delivery used in the emergency department, patient age, the Emergency Severity Index level, respiratory rate, serum lactate, and demographic characteristics. In another study, Haimovich AD et al., showed that a bedside ML model (quick COVID-19 Severity Index) that employed 3 variables (respiratory rate, pulse oximetry, and oxygen flow rate), the COVID-19 Severity Index can be used to predict critical respiratory illness in COVID-19 patients (56). These models outperformed the quick Sequential [Sepsis-related] Organ Failure Assessment, CURB-65 and Elixhauser scores. Furthermore, another study showed that ML models (Neural Network, Random Forest, and Classification and Regression Decision Tree) outperformed conventional tools, including the APACHE II score in predicting critical COVID-19 based on clinical parameters on admission (57).

#### Different clinical settings

Kim J et al., proposed an artificial intelligence model to predict acute respiratory failure 1 h, 2 h, 4 h, and 6 h prior to its occurrence using physiological signatures and past medical history (17). The AUC of this model was 0.869 for respiratory failure 6 h before occurrence. Additionally, the model outperformed the MEWS and NEWS scores (17). Xia M et al., used supervised ML algorithms to predict hypoxemia after extubation in the ICU (58). The authors found that from the tested algorithms (logistic regression, random forest, K-nearest neighbors, support-vector machine, XGBoost, Light Gradient Boosting Machine (LightGBM)), random forest, and Light Gradient Boosting Machine showed the best performance in hypoxemia prediction (58).

ML techniques have been used to predict intubation within 24 h using commonly available bedside and laboratory variables taken at critical care admission. Random forest and logistic regression exhibited good performance for intubation prediction (AUC = 0.86 and 0.77 respectively) (59). Recurrent Neural Network models have been developed to predict the failure of noninvasive respiratory support using time series data (60). The authors showed that a Long-short term memory model had the highest accuracy and AUC compared to a Gated Recurrent Unit and a Gated Recurrent Unit with Trainable Decay (60). In another study, an ML (CatBoost) model was developed to predict noninvasive ventilation failure after extubation (61); fifteen parameters (mechanical ventilation duration, RR, urine output, GCS, mean airway pressure, temperature, age, heart rate, glucose, time from extubation to NIV, mean blood pressure, input volume, SpO2, PaO2, and pH) were used as inputs. The authors showed that the proposed model showed better performance compared to the RF, LR, XGBoost, KNN, Naïve Bayes, Light GBM, SCM, AdaBoost, and MLP (61). Furthermore, a temporal convolutional network-feedforward neural network outperformed the LSTM, feedforward neural networks, and logistic regression in predicting intubation in the critical care setting (62).

An ML algorithm has been used to predict reintubation, prolonged mechanical ventilation and death in patients undergoing coronary artery bypass surgery (63). Specifically, an artificial neural network showed good performance in predicting these outcomes, with no difference compared to the logistic regression model (63). Another novel model for predicting intubation in critically ill patients (64), using data collected within the first hours of admission in the ICU, outperformed the standard clinical benchmarks (64). Recently, a real-time warning algorithm for the prediction of invasive mechanical ventilation in ICU patients was developed (65). The proposed algorithm used seven ML models (LightGBM, Random Forest, Naive Bayes, Neural Networks, Logistic regression, Support Vector Machines, K-Nearest Neighbor), exhibiting improved performance compared to traditional adjustment risk algorithms (65). Interestingly, the model using only non-invasive parameters provided excellent predictive performance, compared to the model using both non-invasive and invasive parameters (65). The Time Updated Light Gradient Boosting Machine model has also been proposed to predict late noninvasive ventilation failure (66), showing better performance in comparison with common models (logistic regression, random forest, LightGBM, XGBoost, artificial neural network, and LSTM) (66).

#### Implications for clinical practice

The integration of AI/ML models into acute care settings carries significant implications for transforming clinical practice, moving towards more proactive and precise patient management.

*Augmented Clinical Decision-Making and Early Intervention* AI/ML models offer a substantial opportunity to augment clinician decision-making, particularly for initial risk stratification and triage in high-volume environments like emergency departments. By providing early warnings of impending CA or RA, these models can broaden the “diagnostic and therapeutic window” for intervention, allowing clinicians to initiate preventive measures well before overt deterioration. This proactive approach represents a marked improvement over current reactive responses, which often occur after a critical event has already begun.

*Potential for Reduced Morbidity and Mortality* The core clinical benefit derived from these models lies in their ability to identify high-risk patients, prompting timely interventions that could significantly reduce in-hospital morbidity and mortality associated with CA/RA. This translates directly to improved patient safety and better overall outcomes, as critical resources and attention can be directed to those most in need, precisely when it matters most.

*Enhanced Monitoring and Proactive Care* The seamless integration of AI/ML with streaming vital signs and EHR can enable continuous, intelligent monitoring. This capability allows for the detection of subtle physiological shifts indicative of worsening disease, often missed by intermittent manual checks. Such a system moves clinical practice from periodic, interval-based assessments to a more dynamic, real-time surveillance system, fostering a culture of pre-emptive care where interventions are initiated before a full-blown crisis develops.

*Necessity of Clinician Education and Workflow Integration* For successful implementation, it is crucial that clinicians receive adequate education on how to effectively use and interpret these AI/ML models, “as labeled”. This implies the need for intuitive user interfaces that present complex AI predictions in an understandable format, clear guidelines on alert interpretation, and thoughtful integration into existing clinical workflows to ensure seamless adoption and avoid disruption to established care processes. Without proper training and integration, even the most accurate models may not achieve their full clinical potential.

*Addressing Regulatory and Ethical Considerations* Prior to widespread clinical adoption, a robust framework must be established to regulate critical issues such as liability for AI-driven decisions, standardized adverse event reporting mechanisms, protocols for system upgrading and maintenance, and stringent cybersecurity measures to protect sensitive patient data. These considerations are foundational for building trust among clinicians and patients and ensuring the responsible and equitable deployment of AI in healthcare.

#### Recommendations for future research

While the potential of AI/ML in acute care is evident, several critical areas require focused future research to facilitate their successful and safe translation into routine clinical practice.

*Rigorous Prospective Validation and Demonstration of Clinical Utility* A paramount recommendation is the urgent need for rigorous prospective evaluation of AI/ML models. While retrospective studies have shown considerable promise, future research must move beyond these to large-scale prospective clinical trials that confirm efficacy in real-world settings. Crucially, these trials must demonstrate a tangible impact on clinical endpoints such as patient mortality, reduced length of stay, or decreased incidence of adverse events. Studies must explicitly show how these approaches translate into “actionable care pathways and workflows” that demonstrate clear clinical utility, rather than merely improved statistical prediction.

*Standardization of Datasets and Platforms* A significant challenge identified is the “lack of uniform datasets and of parameters employed by the proposed AI/ML algorithms”, which currently hinders the assessment of their generalizability and comparability across different institutions. Future research should focus on developing standardized data collection protocols and creating standardized platforms for reporting predictions to clinicians, ensuring interoperability and facilitating broader adoption. Such standardization would enable more robust multi-center studies and foster a collaborative environment for AI development and validation.

*Improving Model Specificity to Mitigate Alarm Burden* While high sensitivity is highly desirable for life-threatening conditions to ensure no critical event is missed, the specificity of a model must also be high for implementation in clinical practice. A low specificity leads to a high burden of false alarms, which can significantly increase clinician workload, induce stress, and potentially lead to alarm fatigue and desensitization. This desensitization could paradoxically result in missed true events, undermining the very goal of patient safety. The inherent tension between maximizing sensitivity (to avoid missing a critical event) and achieving high specificity (to minimize false alarms) in life-threatening conditions presents a profound ethical and practical dilemma for AI in healthcare. Clinicians are ethically bound to prioritize patient safety, meaning they will naturally lean towards higher sensitivity in predictive tools for conditions like cardiac or RA. However, the consequence of high sensitivity without commensurate specificity is an increased rate of false positives. A high burden of false alarms results in increased workload and stress for healthcare providers and eventually alarm fatigue. Prioritizing the optimization of the balance between sensitivity and specificity to ensure practical utility and avoid clinician burnout necessitates interdisciplinary research involving not just AI developers but also human factors specialists, such as clinicians and healthcare administrators, to design systems that are both statistically effective and clinically usable, perhaps through adaptive alerting systems or tiered alert levels.

*Addressing Data Quality, Noise, and Ground-Truth Labeling* Real-world clinical data often suffer from “noise” and variability in quality, with some studies reporting valid data for as little as half of the monitoring time. Future research must develop robust methods for handling incomplete or noisy data to ensure model reliability in diverse clinical environments. Furthermore, accurate “ground-truth labels” are fundamental for effective AI/ML algorithm training, and current methods like natural language processing for label generation can be prone to errors, while semi-supervised models remain in the research phase.

*Ethical AI Development and Governance* Beyond technical performance, future AI/ML models must be developed with explicit consideration of ethical principles, including equity, accuracy, transparency, interpretability, accountability, data privacy, and cybersecurity (32, 33). These considerations are not merely regulatory hurdles but foundational requirements for building trust and ensuring the responsible and equitable integration of AI into clinical care. *Furthermore, r*esearch into explainable AI and fairness in algorithms will be crucial to address these concerns.

*Larger Sample Sizes and Generalizability* The current body of evidence largely comprises studies with “relatively small sample sizes”, which limits the generalizability of their findings. Future research must prioritize larger-scale, multi-center studies to validate model performance across diverse patient populations and clinical environments, ensuring robust and generalizable results that can be applied broadly.

*Systemic Redesign for Actionable Care Pathways* The repeated emphasis on the need for AI models to translate into “actionable care pathways and workflows” signifies that the objective extends far beyond merely developing a technically superior predictive algorithm. An AI model, no matter how accurate, is an inert tool if its predictions do not seamlessly integrate into and actively inform clinical decision-making and subsequent actions. This implies a need for a fundamental redesign of existing clinical processes, rather than simply overlaying AI on top of current practices. For example, an early warning from an AI system must trigger a predefined, efficient, and well-rehearsed response protocol involving specific roles, responsibilities, and interventions. This necessitates interdisciplinary research and development involving not only AI specialists but also clinical workflow experts, engineers, healthcare administrators, and even policy-makers. The ultimate success of AI in healthcare will hinge on its ability to catalyze and support these systemic changes, transforming predictive insights into tangible improvements in patient care delivery and outcomes.

## Discussion

In-hospital CA and RA are catastrophic complications of any admission. It is estimated that between 1 and 5 of every 1,000 admissions yearly will result in CA and RA (67), while the survival rate for in-hospital CA remains between 23% and 24% (2, 3, 68). However, efforts to develop early warning scores of deterioration aiming to activate rapid response protocols (6–11), should recognize that there is only a limited time-window to provide pre-emptive care. Retrospective reviews frequently show that signs of deterioration are unobserved or overlooked by medical staff (12, 13). Continuous telemetry monitoring is routine in the ICU and some non-ICU units (69, 70), yet CAs and RAs are still frequent.

To assess whether current developments on ML models can improve outcomes in predicting CA and RA, a systematic search of PubMed, Embase, and Web of Science was conducted. The search strategy focused on critical care settings, AI/ML techniques, and cardiac or RA outcomes. The selection process is detailed in Figures 2A,B, resulting in 14 CA and 22 RA studies included for analysis.

**Figure 3:**
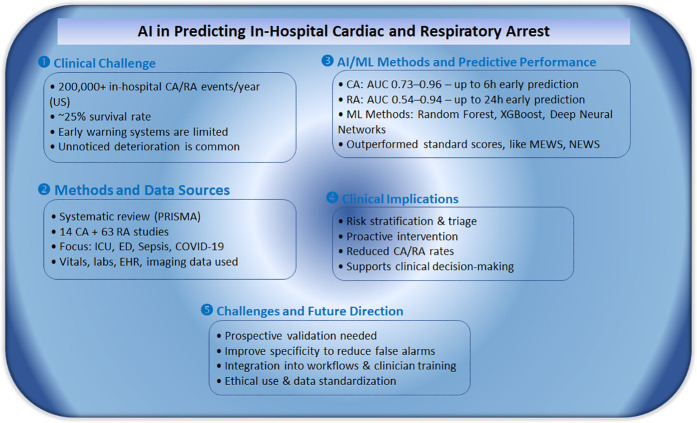
Summary of key findings from a systematic review on AI-based prediction of in-hospital cardiac and respiratory arrest.

Improving not just survival but also the quality of care for in-hospital CA patients requires a comprehensive set of programs and actions, such as, *first*, plans and preparation for CA and RA, *second*, delivery of high-quality, guideline-based resuscitation, *third*, continuous evaluation and improvement itself within a culture of person-centered care, and *fourth*, the potential for AI to assist in the prediction and prevention of CA. Although the prediction of cardiac and RA could reduce in-hospital morbidity and mortality, further studies are needed to confirm this in clinical practice. Identification of high-risk patients especially in the emergency department is of great importance (Figure 3). Furthermore, enhanced monitoring and early preventive measures may help identify high-risk hospitalized patients, prevent adverse clinical outcomes, and thus reduce morbidity and mortality. This systematic review shows that ML models may be used for the prediction of both cardiac and RA in the emergency department and in the ICU. Furthermore, the retrospective studies show that the proposed models have a good prediction performance using easily obtained variables. Interestingly, in the prospective studies, although it is not clearly mentioned, the results of the AI/ML prediction models were not shared with the attending physicians, and therefore they did not influence clinical outcomes.

While ML algorithms show a promising performance in predicting in-hospital cardiac and RA, the integration of these models into clinical workflows remains a significant challenge. Practical considerations include integration with the electronic health record systems, ensuring data interoperability, and adequate staff training to effectively utilize the predictions from these models to improve clinical-decision outcomes. However, further research is needed to understand the real-world barriers to designing and implementing ML tools in clinical practice.

## Limitations

Most of the included studies were of relatively small sample size, and therefore the results should be interpreted with caution. There was also substantial heterogeneity across studies in terms of study design, ML methodologies, and data sources, which may affect the comparability and generalizability of the results. In clinical practice, the quality of data that are required as inputs cannot be identical. Although AI systems have been shown to improve accuracy over traditional diagnostic systems, albeit with a broad range of accuracy, prospective studies on the clinical validation of these models for forecasting clinical deterioration are important, yet they are relatively sparse. The specificity of a model must be high for implementation in clinical practice. A low specificity will lead in a high burden of false alarms that will increase the workload and stress of healthcare providers. Furthermore, prospective studies are needed not only to further establish the accuracy and generalizability of these approaches, but also their translation to actionable care pathways, which can demonstrate clinical utility.

## Conclusions

ML algorithms show promising results for the prediction of in-patient cardiac and RA using easily obtained variables as inputs. If successfully implemented in clinical practice, the ML models could identify high-risk patients and reduce mortality and morbidity. However, further validation and the design of clinical trials will determine the efficacy of the ML models in each clinical setting.

## Data Availability

The original contributions presented in the study are included in the article/Supplementary Material, further inquiries can be directed to the corresponding author.
