# Supplementary material for: Use of artificial intelligence in predicting in-hospital cardiac and respiratory arrest in an acute care environment—implications for clinical practice
Source: Front Med Technol. 2025 Oct 10;7:1681059. doi: 10.3389/fmedt.2025.1681059 (PMC12549753; doi:10.3389/fmedt.2025.1681059)
Supplement: Supplementary file 1 [file Table1.docx]

**Supplementary Table 1. Quality assessment of the included studies**

N positive responses: indicates the sum of “yes” responses in the different quality assessment categories in each study.

| **Author** | **Unmet Need** | **Reproducibility** | | | **Robustness** | | **Generalizability** | **Clinical Significance** | | N  positive responses |
| --- | --- | --- | --- | --- | --- | --- | --- | --- | --- | --- |
|  | Limits in current non-machine-learning approach | Feature engineering methods | Platforms/packages | Hyperparameters | Valid methods to overcome over-fit | Stability of results | External data validation | Predictors explanation | Suggested clinical use |  |
| **Respiratory arrest** | | | | | | | | | | |
| Bendavid I (1) | Yes | Yes | No | Yes | No | Yes | No | Yes | No | 5 |
| Siu BMK (2) | Yes | Yes | No | Yes | Yes | Yes | Yes | Yes | No | 7 |
| Bolourani S (3) | Yes | Yes | No | Yes | Yes | No | No | No | Yes | 5 |
| Heldt FS (4) | Yes | Yes | No | Yes | No | No | No | Yes | No | 4 |
| Haimovich AD (5) | Yes | Yes | No | Yes | Yes | No | No | Yes | Yes | 6 |
| Boussen S (6) | Yes | Yes | No | No | No | No | No | No | Yes | 3 |
| Karri R (7) | Yes | Yes | No | Yes | No | No | No | Yes | Yes | 5 |
| Assaf D (8) | Yes | Yes | No | No | No | No | No | No | No | 2 |
| Shashikumar SP (9) | Yes | Yes | No | Yes | Yes | Yes | No | No | Yes | 6 |
| Wang H (10) | Yes | No | No | Yes | No | No | No | Yes | Yes | 4 |
| Kim J (11) | Yes | No | No | No | No | Yes | No | No | Yes | 3 |
| Xia M (12) | Yes | Yes | No | Yes | Yes | Yes | No | Yes | Yes | 7 |
| Essay PT (13) | Yes | No | No | Yes | No | Yes | No | No | Yes | 4 |
| Wang H (14) | Yes | Yes | Yes | Yes | Yes | Yes | Yes | Yes | Yes | 9 |
| Di Napoli A (15) | Yes | Yes | No | Yes | Yes | No | Yes | No | Yes | 6 |
| Chamberlin JH (16) | Yes | Yes | Yes | No | Yes | No | Yes | No | Yes | 6 |
| Yu L (17) | Yes | Yes | No | Yes | Yes | No | No | Yes | Yes | 6 |
| Catling FJR (18) | Yes | Yes | No | Yes | Yes | No | No | Yes | Yes | 6 |
| Mendes RG (19) | Yes | Yes | No | No | No | No | No | No | Yes | 3 |
| Venturini M (20) | Yes | Yes | No | No | No | No | No | No | No | 2 |
| Feng X (21) | Yes | Yes | No | Yes | Yes | No | No | Yes | Yes | 6 |
| de Godoy MF (22) | Yes | Yes | No | No | Yes | Yes | No | No | Yes | 5 |
| **Cardiac Arrest** | | | | | | | | | | |
| Yijing L (23) | Yes | Yes | No | No | Yes | Yes | No | Yes | Yes | 5 |
| Kim J (24) | Yes | Yes | No | No | Yes | Yes | No | No | Yes | 5 |
| Lu TC (25) | Yes | Yes | No | Yes | Yes | Yes | No | Yes | Yes | 7 |
| Kim J (11) | Yes | Yes | No | No | No | No | No | No | Yes | 3 |
| Tang Q (26) | Yes | No | No | No | Yes | No | No | Yes | No | 3 |
| Layeghian Javan S (27) | Yes | Yes | No | Yes | Yes | No | No | No | No | 4 |
| Wu TT (28) | Yes | Yes | No | No | No | No | No | No | No | 2 |
| Ong ME (29) | Yes | No | No | No | Yes | No | No | No | No | 2 |
| Jang DH (30) | Yes | Yes | No | Yes | No | No | No | No | No | 3 |
| Kim JH (31) | Yes | Yes | No | Yes | Yes | No | No | Yes | Yes | 6 |
| Chen MC (32) | Yes | Yes | No | No | Yes | No | No | No | No | 3 |
| Lee H (33) | Yes | No | No | No | No | No | No | No | No | 1 |
| Nan Liu (34) | Yes | Yes | No | No | Yes | No | No | Yes | No | 4 |
| Baral S (35) | Yes | Yes | No | Yes | No | No | No | No | Yes | 4 |

**References**

1. Bendavid I, Statlender L, Shvartser L, Teppler S, Azullay R, Sapir R, et al. A novel machine learning model to predict respiratory failure and invasive mechanical ventilation in critically ill patients suffering from COVID-19. Sci Rep. 2022;12(1):10573.

2. Siu BMK, Kwak GH, Ling L, Hui P. Predicting the need for intubation in the first 24 h after critical care admission using machine learning approaches. Sci Rep. 2020;10(1):20931.

3. Bolourani S, Brenner M, Wang P, McGinn T, Hirsch JS, Barnaby D, et al. A Machine Learning Prediction Model of Respiratory Failure Within 48 Hours of Patient Admission for COVID-19: Model Development and Validation. J Med Internet Res. 2021;23(2):e24246.

4. Heldt FS, Vizcaychipi MP, Peacock S, Cinelli M, McLachlan L, Andreotti F, et al. Early risk assessment for COVID-19 patients from emergency department data using machine learning. Sci Rep. 2021;11(1):4200.

5. Haimovich AD, Ravindra NG, Stoytchev S, Young HP, Wilson FP, van Dijk D, et al. Development and Validation of the Quick COVID-19 Severity Index: A Prognostic Tool for Early Clinical Decompensation. Ann Emerg Med. 2020;76(4):442-53.

6. Boussen S, Cordier PY, Malet A, Simeone P, Cataldi S, Vaisse C, et al. Triage and monitoring of COVID-19 patients in intensive care using unsupervised machine learning. Comput Biol Med. 2022;142:105192.

7. Karri R, Chen YP, Burrell AJC, Penny-Dimri JC, Broadley T, Trapani T, et al. Machine learning predicts the short-term requirement for invasive ventilation among Australian critically ill COVID-19 patients. PLoS One. 2022;17(10):e0276509.

8. Assaf D, Gutman Y, Neuman Y, Segal G, Amit S, Gefen-Halevi S, et al. Utilization of machine-learning models to accurately predict the risk for critical COVID-19. Intern Emerg Med. 2020;15(8):1435-43.

9. Shashikumar SP, Wardi G, Paul P, Carlile M, Brenner LN, Hibbert KA, et al. Development and Prospective Validation of a Deep Learning Algorithm for Predicting Need for Mechanical Ventilation. Chest. 2021;159(6):2264-73.

10. Wang H, Wang C, Xu J, Yuan J, Liu G, Zhang G. Invasive mechanical ventilation probability estimation using machine learning methods based on non-invasive parameters. Biomedical Signal Processing and Control. 2023;79:104193.

11. Kim J, Chae M, Chang HJ, Kim YA, Park E. Predicting Cardiac Arrest and Respiratory Failure Using Feasible Artificial Intelligence with Simple Trajectories of Patient Data. J Clin Med. 2019;8(9).

12. Xia M, Jin C, Cao S, Pei B, Wang J, Xu T, et al. Development and validation of a machine-learning model for prediction of hypoxemia after extubation in intensive care units. Ann Transl Med. 2022;10(10):577.

13. Essay PT, Mosier JM, Nayebi A, Fisher JM, Subbian V. Predicting Failure of Noninvasive Respiratory Support Using Deep Recurrent Learning. Respir Care. 2023;68(4):488-96.

14. Wang H, Zhao QY, Luo JC, Liu K, Yu SJ, Ma JF, et al. Early prediction of noninvasive ventilation failure after extubation: development and validation of a machine-learning model. BMC Pulm Med. 2022;22(1):304.

15. Di Napoli A, Tagliente E, Pasquini L, Cipriano E, Pietrantonio F, Ortis P, et al. 3D CT-Inclusive Deep-Learning Model to Predict Mortality, ICU Admittance, and Intubation in COVID-19 Patients. J Digit Imaging. 2023;36(2):603-16.

16. Chamberlin JH, Aquino G, Schoepf UJ, Nance S, Godoy F, Carson L, et al. An Interpretable Chest CT Deep Learning Algorithm for Quantification of COVID-19 Lung Disease and Prediction of Inpatient Morbidity and Mortality. Acad Radiol. 2022;29(8):1178-88.

17. Yu L, Halalau A, Dalal B, Abbas AE, Ivascu F, Amin M, et al. Machine learning methods to predict mechanical ventilation and mortality in patients with COVID-19. PLoS One. 2021;16(4):e0249285.

18. Catling FJR, Wolff AH. Temporal convolutional networks allow early prediction of events in critical care. J Am Med Inform Assoc. 2020;27(3):355-65.

19. Mendes RG, de Souza CR, Machado MN, Correa PR, Di Thommazo-Luporini L, Arena R, et al. Predicting reintubation, prolonged mechanical ventilation and death in post-coronary artery bypass graft surgery: a comparison between artificial neural networks and logistic regression models. Arch Med Sci. 2015;11(4):756-63.

20. Venturini M, Keilegom IV, Corte WD, Vens C. A Novel Survival Analysis Approach to Predict the Need for Intubation in Intensive Care Units. Artificial Intelligence in Medicine: 20th International Conference on Artificial Intelligence in Medicine, AIME 2022, Halifax, NS, Canada, June 14–17, 2022, Proceedings; Halifax, NS, Canada: Springer-Verlag; 2022. p. 358–64.

21. Feng X, Pan S, Yan M, Shen Y, Liu X, Cai G, et al. Dynamic prediction of late noninvasive ventilation failure in intensive care unit using a time adaptive machine model. Comput Methods Programs Biomed. 2021;208:106290.

22. de Godoy MF, Chatkin JM, Rodrigues RS, Forte GC, Marchiori E, Gavenski N, et al. Artificial intelligence to predict the need for mechanical ventilation in cases of severe COVID-19. Radiol Bras. 2023;56(2):81-5.

23. Yijing L, Wenyu Y, Kang Y, Shengyu Z, Xianliang H, Xingliang J, et al. Prediction of cardiac arrest in critically ill patients based on bedside vital signs monitoring. Comput Methods Programs Biomed. 2022;214:106568.

24. Kim J, Park YR, Lee JH, Lee JH, Kim YH, Huh JW. Development of a Real-Time Risk Prediction Model for In-Hospital Cardiac Arrest in Critically Ill Patients Using Deep Learning: Retrospective Study. JMIR Med Inform. 2020;8(3):e16349.

25. Lu TC, Wang CH, Chou FY, Sun JT, Chou EH, Huang EP, et al. Machine learning to predict in-hospital cardiac arrest from patients presenting to the emergency department. Intern Emerg Med. 2023;18(2):595-605.

26. Tang Q, Cen X, Pan C. Explainable and efficient deep early warning system for cardiac arrest prediction from electronic health records. Math Biosci Eng. 2022;19(10):9825-41.

27. Layeghian Javan S, Sepehri MM, Layeghian Javan M, Khatibi T. An intelligent warning model for early prediction of cardiac arrest in sepsis patients. Comput Methods Programs Biomed. 2019;178:47-58.

28. Wu TT, Zheng RF, Lin ZZ, Gong HR, Li H. A machine learning model to predict critical care outcomes in patient with chest pain visiting the emergency department. BMC Emerg Med. 2021;21(1):112.

29. Ong ME, Lee Ng CH, Goh K, Liu N, Koh ZX, Shahidah N, et al. Prediction of cardiac arrest in critically ill patients presenting to the emergency department using a machine learning score incorporating heart rate variability compared with the modified early warning score. Crit Care. 2012;16(3):R108.

30. Jang DH, Kim J, Jo YH, Lee JH, Hwang JE, Park SM, et al. Developing neural network models for early detection of cardiac arrest in emergency department. Am J Emerg Med. 2020;38(1):43-9.

31. Kim JH, Choi A, Kim MJ, Hyun H, Kim S, Chang HJ. Development of a machine-learning algorithm to predict in-hospital cardiac arrest for emergency department patients using a nationwide database. Sci Rep. 2022;12(1):21797.

32. Chen MC, Huang TY, Chen TY, Boonyarat P, Chang YC. Clinical narrative-aware deep neural network for emergency department critical outcome prediction. J Biomed Inform. 2023;138:104284.

33. Lee H, Shin SY, Seo M, Nam GB, Joo S. Prediction of Ventricular Tachycardia One Hour before Occurrence Using Artificial Neural Networks. Sci Rep. 2016;6:32390.

34. Liu N, Koh ZX, Goh J, Lin Z, Haaland B, Ting BP, et al. Prediction of adverse cardiac events in emergency department patients with chest pain using machine learning for variable selection. BMC Med Inform Decis Mak. 2014;14:75.

35. Baral S, Alsadoon A, Prasad PWC, Al Aloussi S, Alsadoon OH. A novel solution of using deep learning for early prediction cardiac arrest in Sepsis patient: enhanced bidirectional long short-term memory (LSTM). Multimedia Tools and Applications. 2021;80(21):32639-64.
