# Supplementary material for: Use of artificial intelligence in predicting in-hospital cardiac and respiratory arrest in an acute care environment—implications for clinical practice
Source: Front Med Technol. 2025 Oct 10;7:1681059. doi: 10.3389/fmedt.2025.1681059 (PMC12549753; doi:10.3389/fmedt.2025.1681059)
Supplement: Supplementary file 2 [file Table2.docx]

**Literature Search Strategy**

A comprehensive search was conducted in PubMed, Embase, and Web of Science using the advanced search builder. The strategy was structured around three major conceptual domains: artificial intelligence and machine learning, critical care, and cardiac or respiratory arrest. Boolean operators “OR” were used to combine synonymous terms within each domain, while “AND” was used to link the three domains together.

For the artificial intelligence domain, the following MeSH terms were included: “Artificial Intelligence,” “Machine Learning,” “Deep Learning,” “Supervised Machine Learning,” “Unsupervised Machine Learning,” “Neural Networks, Computer,” and “Random Forest.” To ensure comprehensive retrieval of relevant literature, the search was escalated by independently including subgroup terms in the Title/Abstract and Other Term fields. These included “artificial intelligence,” “machine learning,” “deep learning,” “transfer learning,” “hierarchical learning,” “supervised machine learning,” “unsupervised machine learning,” “semi supervised machine learning,” “neural network,” “neural networks, computer,” “deep neural network,” “recurrent neural network,” “random forest,” and “Bayesian learning.”

For the critical care domain, MeSH terms included “Critical Illness,” “Critical Care,” “Intensive Care Units,” “Coronary Care Units,” and “Respiratory Care Units.” Subgroup terms were also searched independently in the Title/Abstract and Other Term fields to capture additional variations and usage in the literature. These included “critical illness,” “critical illnesses,” “critically ill,” “critical care,” “intensive care,” “intensive care unit,” “intensive care units,” “coronary care unit,” “coronary care units,” “respiratory care unit,” and “respiratory care units.”

The cardiac and respiratory arrest domain incorporated the following MeSH terms: “Respiratory Insufficiency,” “Respiration, Artificial,” “Airway Management,” “Intubation, Intratracheal,” “Heart Arrest,” “Cardiopulmonary Arrest,” “Death, Sudden, Cardiac,” and “Ventricular Fibrillation.” To enhance sensitivity, additional related terms were searched independently in the Title/Abstract and Other Term fields, including “respiratory insufficiency,” “respiratory failure,” “respiratory failures,” “respiratory depression,” “ventilatory depression,” “hypoxemic respiratory failure,” “artificial respiration,” “mechanical ventilation,” “respiratory arrest,” “airway management,” “intratracheal intubation,” “endotracheal intubation,” “lung insufficiency,” “invasive ventilation,” “heart arrest,” “cardiac arrest,” “asystole,” “asystoles,” “cardiopulmonary arrest,” “sudden cardiac death,” “cardiac sudden death,” “sudden cardiac arrest,” “ventricular fibrillation,” “ventricular fibrillations,” and “sudden arrhythmic death syndrome.”

The final search string followed the structure: (artificial intelligence and machine learning terms) AND (critical care terms) AND (cardiac and respiratory arrest terms).

**Fundamentals of AI Algorithms Reported in this Manuscript**

***Gradient Boosting*** In gradient boosting (GB), the process begins with the initial model that trains on the raw input data and makes predictions. The residuals, the difference between actual and predicted values are calculated. The second model is trained on these residuals, effectively learning to correct the errors made by the first model. This process continues iteratively, with each subsequent model being trained on the residuals of the combined predictions of all previous models, and updating the predictions accordingly. Thus GB algorithm utilizes sequential learning, progressively combining weak learners to form a stronger ensemble[2].

***Extreme Gradient Boosting*** Extreme Gradient boosting (XGBoost) is an advanced and optimized version of gradient boosting which incorporates the several features to the GB algorithm. The XGBoost algorithm uses the second-order approximation in gradient-descent and includes both the gradient and Hessian (second derivative), L1(Lasso) and L2(Ridge) regularization and scalability supporting Parallel processing, GPU acceleration etc to efficiently handle the large datasets with lesser computation time[1].

***Light Gradient Boosting(LGB)*** The Light Gradient Boosting Machine (LightGBM) algorithm is very similar to the XGBoost algorithm. However, LightGBM uses histogram-based binning methods for tree growth, which results in faster execution and reduced memory usage. It also focuses on leaf-wise splitting, as opposed to the level-wise splitting used in the XGBoost algorithm[2].

***Catboost* CatBoost** (Categorical Boosting) is a variant of gradient boosting which can handle both categorical and numerical features. It does not require feature encoding techniques for categorical data. Catboost can process these features directly. It employs an ordered boosting principle, which helps reduce overfitting by using permutations of the data to build more robust models[3].

***Adaboost* AdaBoost** is a boosting algorithm that combines multiple weak learners to create a strong classifier. It works by updating the weights of the instances, increasing the weights of those that were misclassified. Each new model is trained to focus on these updated weights, which emphasizes the misclassified instances from previous models and improves the overall performance[4].

***Decision Tree*** A Decision Tree is a supervised learning algorithm that splits data into subsets based on feature values to make predictions. Each split is chosen to best separate the data based on some criteria, typically aiming to maximize information gain, or minimize impurity[5].

***CRT*** A specific type of implementation of decision trees for classification and regression task. The best decision split is decided by the measure such as Gini impurity for classification and measures such as mean squared error for regression for the split criteria[6].

***Random Forest*** Random Forest is an ensemble method that combines the predictions from multiple decision trees trained on different subsets of data. The aggregation of predictions from these decision trees is based on the bagging principle. Bootstrap aggregating (bagging) involves using majority voting for classification tasks and averaging for regression tasks[7].

***Extra Trees Classifier*** The Extra Trees Classifier is an ensemble method similar to Random Forest, but with more randomness introduced during tree construction. Both methods build multiple decision trees and use a random subset of features for each split. However, Extra Trees further randomizes the process by selecting split points randomly rather than optimizing them based on some optimization criteria(Gini-impurity, entropy for classification and mean-square-error for regression), which increases tree diversity and can reduce overfitting[8].

***Artificial Neural Networks*** Artificial Neural Networks (ANNs) are inspired by the human brain to perform complex tasks. These networks consist of input, hidden, and output layers. The input layer receives raw data, while the output layer provides the final result based on computations. The hidden layers, which can be ‘n’ number of layers, process the input using weights and biases. Activation functions in the neurons are used to introduce non-linearity to the model. To train the network, the backpropagation algorithm is used in conjunction with gradient descent. This learning process involves updating the weights of the neurons to improve performance. Networks with more than two layers are referred to as deep neural networks, whereas those with only one layer are called shallow neural networks[9].

***Multi-Layer Perceptron*** A Multi-Layer Perceptron (MLP) is a specific type of feedforward artificial neural network characterized by its fully connected layers. In an MLP, information flows in only one direction, from the input layer to the output layer. It consists of one or more hidden layers of neurons between the input and output layers. Activation functions such as tanh, ReLU, and sigmoid are used to introduce non-linearity into the model. The network is trained using backpropagation to update the weights and gradient descent to minimize the loss function[10].

***Logistic Regression*** Logistic Regression is a supervised machine learning algorithm that uses the logistic function (also known as the sigmoid function) to model the probability of a binary outcome. It predicts the likelihood of an input belonging to a particular class by transforming a linear combination of input features into a probability value between 0 and 1[11].

***Ridge Regression*** Ridge Regression is an extension of linear regression that includes L2 regularization to address overfitting. It modifies the standard least squares loss function by adding a penalty proportional to the square of the magnitude of the model coefficients. This penalty shrink the coefficients, which further reduce both overfitting and multicollinearity issues[12].

***XGBoost and SMOTEENN*** It uses the XGBoost algorithm along with the technique that combines SMOTE (Synthetic Minority Over-sampling Technique) and Edited Nearest Neighbors to address class imbalance by generating synthetic samples and removing the irrelevant samples from the dataset.

***Time Updated Light Gradient Boosting Machine*** The TULightGBM model uses multiple LightGBM classifiers, each trained on different time periods of clinical data to capture varying aspects of a patient's condition over time. These classifiers make predictions for each time segment, which are then combined by a dynamic attention mechanism. This mechanism assigns weights to the predictions, allowing the model to adaptively update and improve predictions as new clinical data becomes available.

***Support Vector Machine* Support Vector Machine (SVM)** is a supervised learning algorithm used for both classification and regression tasks. For classification, SVM aims to find the optimal hyperplane that maximizes the margin (the distance) between two classes. This hyperplane effectively separates the two groups in the feature space. It also uses the kernel functions to handle the non-linear data. For regression, SVM tries to find the best line (or hyperplane) that has maximum data points[13].

***K-Nearest Neighbors* K-Nearest Neighbors (KNN)** is a supervised learning algorithm used for classification and regression. In KNN, similar data points are located near each other based on their proximity in the feature space. To classify a new data point, KNN looks at the k nearest points in the training dataset and determines the most common class (for classification) or the average value (for regression) among these neighbors. KNN works with distance metrics such as Euclidean distance and Manhattan distance to measure proximity between points[14].

***Gaussian Naive Bayes* Gaussian Naive Bayes (Gaussian NB)** is a classification algorithm that assumes features follow a Gaussian (normal) distribution within each class. It calculates the likelihood of a data point’s features under this Gaussian assumption, estimating the mean and variance for each class. By applying Bayes' theorem, Gaussian NB computes posterior probabilities for each class and assigns the class with the highest probability[15].

***Clustering* Clustering** is an unsupervised learning technique where data points are grouped into clusters or groups based on their similarities, without prior knowledge of labels. Data points within the same cluster belong to the same group and are more similar to each other than to points in other clusters. Common clustering algorithms include K-Means, Hierarchical Clustering, and DBSCAN. The clustering can be evaluated using metrics such as the within-cluster sum of squares (WCSS), silhouette score, which measure how well the clusters distinct they are from one another[16].

***Temporal Convolutional Neural Network - Feedforward Neural Network*** The **TCN-FFNN** model integrates a Temporal Convolutional Network (TCN) with a Feedforward Neural Network (FFNN) to handle both longitudinal and demographic data. The TCN processes sequential data to capture temporal patterns, while the FFNN processes static demographic features with dropout for regularization. The outputs of both networks are concatenated and fed into separate binary logistic regression models to generate final predictions[17].

***Long Short-Term Memory*** Long Short-Term Memory (LSTM) networks are a type of recurrent neural network (RNN) designed to process and analyze sequential data, such as text, time-series, and speech data. They use gates to control the flow of information and manage long-range dependencies within the data. This gating mechanism helps LSTMs effectively retain and utilize information over extended sequences, addressing the issue of vanishing gradients that affect traditional RNNs[18].

**References**

1. Chen, T. and C. Guestrin, *XGBoost: A Scalable Tree Boosting System*, in *Proceedings of the 22nd ACM SIGKDD International Conference on Knowledge Discovery and Data Mining*2016, Association for Computing Machinery: San Francisco, California, USA. p. 785–794.

2. Ke, G., et al., *LightGBM: a highly efficient gradient boosting decision tree*, in *Proceedings of the 31st International Conference on Neural Information Processing Systems*2017, Curran Associates Inc.: Long Beach, California, USA. p. 3149–3157.

3. Prokhorenkova, L., et al., *CatBoost: unbiased boosting with categorical features*, in *Proceedings of the 32nd International Conference on Neural Information Processing Systems*2018, Curran Associates Inc.: Montréal, Canada. p. 6639–6649.

4. Wang, R., *AdaBoost for Feature Selection, Classification and Its Relation with SVM, A Review.* Physics Procedia, 2012. **25**: p. 800-807.

5. Song, Y.Y. and Y. Lu, *Decision tree methods: applications for classification and prediction.* Shanghai Arch Psychiatry, 2015. **27**(2): p. 130-5.

6. Ozcan, M. and S. Peker, *A classification and regression tree algorithm for heart disease modeling and prediction.* Healthcare Analytics, 2023. **3**: p. 100130.

7. Breiman, L., *Random Forests.* Machine Learning, 2001. **45**(1): p. 5-32.

8. Geurts, P., D. Ernst, and L. Wehenkel, *Extremely randomized trees.* Machine Learning, 2006. **63**(1): p. 3-42.

9. Han, S.H., et al., *Artificial Neural Network: Understanding the Basic Concepts without Mathematics.* Dement Neurocogn Disord, 2018. **17**(3): p. 83-89.

10. Gardner, M.W. and S.R. Dorling, *Artificial neural networks (the multilayer perceptron)—a review of applications in the atmospheric sciences.* Atmospheric Environment, 1998. **32**(14): p. 2627-2636.

11. LaValley, M.P., *Logistic Regression.* Circulation, 2008. **117**(18): p. 2395-2399.

12. McDonald, G.C., *Ridge regression.* WIREs Computational Statistics, 2009. **1**(1): p. 93-100.

13. Cervantes, J., et al., *A comprehensive survey on support vector machine classification: Applications, challenges and trends.* Neurocomputing, 2020. **408**: p. 189-215.

14. Guo, G., et al. *KNN Model-Based Approach in Classification*. 2003. Berlin, Heidelberg: Springer Berlin Heidelberg.

15. Rish, I., *An Empirical Study of the Naïve Bayes Classifier.* IJCAI 2001 Work Empir Methods Artif Intell, 2001. **3**.

16. Li, Y. and H. Wu, *A Clustering Method Based on K-Means Algorithm.* Physics Procedia, 2012. **25**: p. 1104-1109.

17. Yamashita, R., et al., *Convolutional neural networks: an overview and application in radiology.* Insights into Imaging, 2018. **9**(4): p. 611-629.

18. Sherstinsky, A., *Fundamentals of Recurrent Neural Network (RNN) and Long Short-Term Memory (LSTM) network.* Physica D: Nonlinear Phenomena, 2020. **404**: p. 132306.
